# Impact of naturally occurring amino acid variations on the detection of HIV-1 p24 in diagnostic antigen tests

**DOI:** 10.1186/s12879-015-1174-7

**Published:** 2015-10-29

**Authors:** Beatrice N. Vetter, Vanessa Orlowski, Christoph Niederhauser, Louise Walter, Jörg Schüpbach

**Affiliations:** Swiss National Center for Retroviruses (SNCR), Institute of Medical Virology, University of Zürich, Zürich, Switzerland; Institute of Molecular Life Science, University of Zürich, Zürich, Switzerland; Blood Transfusion Service, Swiss Red Cross Berne (BSDSRK), Berne, Switzerland; Official Medicines Control Laboratory, Biologika, Swissmedic, Berne, Switzerland

**Keywords:** HIV, p24, Diagnostic tests, Amino acid, Escape, Detection

## Abstract

**Background:**

The detection of HIV-1 p24 antigen in diagnostic tests relies on antibodies binding to conserved areas of the protein to cover the full range of HIV-1 subtypes. Using a panel of 43 different virus-like particles (VLPs) expressing Gag from clinical HIV-1 isolates, we previously found that some highly sensitive tests completely failed to detect p24 of certain VLPs, seemingly unrelated to their subtype. Here we aimed to investigate the reason for this failure, hypothesising that it might be due to single amino acid variations in conserved epitopes.

**Methods:**

Using amino acid alignment, we identified single amino acid variations at position 16 or 170 of p24, unique to those VLPs that failed to be detected in certain diagnostic tests. Through DNA-mutagenesis, these amino acids were changed to ones more commonly found at these positions. The impact of these changes on p24 detection was tested in commercial diagnostic tests as well as by Western Blot and ELISA, using epitope-specific antibodies.

**Results and Conclusions:**

Changing positions 16 or 170 to consensus amino acids restored the detection of p24 by the investigated diagnostic tests as well as by epitope-specific antibodies in Western Blot and ELISA. Hence, single amino acid changes in conserved epitopes can lead to the failure of p24 detection and thus to false-negative results. To optimise HIV diagnostic tests, they should also be evaluated using isolates which harbour less-frequent epitope variants.

## Background

In HIV diagnostics, immunoassays for the detection of viral capsid protein p24 are employed at the stage of screening and diagnosis [1]. In the so-called 4^th^ generation combination screening tests, p24 is used to identify patients with primary infection, prior to the development of anti-HIV antibodies (seroconversion). Technically, the viral antigen is detected in a sandwich format, employing antibodies binding to distinct regions of the protein for capture and detection. These antibodies should bind to highly conserved regions of the target protein, thus minimizing the risk of reduced antigen sensitivity due to subtype-dependent sequence variability or evolutionary escape, both potentially leading to false-negative results. Even single amino acid variations can suffice to decrease the analytical sensitivity of diagnostic immunoassays, as demonstrated for vaccine-induced variants of hepatitis B surface antigen [2, 3]. In the case of HIV, no such diagnostic escape variants have been described for p24, most likely because they simply go unnoticed: An acute primary infection would be false-negative in a 4^th^ generation screening test and thus not detected unless an HIV RNA nucleic acid test was performed in parallel, and a chronic infection would be detected via a patient’s antibody response.

Manufacturers of diagnostic tests evaluate sequence-associated test sensitivity by testing HIV strains from different subtypes and frequent circulating recombinant forms (CRFs), thus hoping to cover a range of sequence variants. However, in a recent study conducted by our laboratory, using 43 virus-like-particles (VLPs) derived from clinical HIV-1 isolates expressing the Gag proteins of different subtypes and CRFs, we noticed that the failure of a diagnostic test to detect p24 of certain virus isolates was not necessarily related to their subtype, as even otherwise highly sensitive tests failed to detect a common subtype B VLP [4]. We hypothesised that detection failure could be due to subtype-independent amino acid variations and analysed our p24 sequences in relation to amino acid variation and detection failure. In this study, we sought to identify amino acid variations that abrogated the detection of p24 in diagnostic HIV-1 p24 antigen-only or 4^th^-generation HIV screening assays, or by epitope-specific antibodies used in home-made tests.

## Methods

### Virus-like particle cloning, production and quantification

VLPs used in this study were expressed from Gag-Pol encoding eukaryotic expression vectors, which were cloned for a previous study conducted in our laboratory [4]. Briefly, the *gag-pr* region of patient-derived viral RNA was cloned into the VLP backbone vector of pCMVΔ8.91, after removing its existing *gag-pr* portion. VLPs were produced by transiently transfecting 293 T cells and VLPs were purified via ultracentrifugation over a 20 % sucrose cushion. VLP quantification and normalisation of concentration was based on reverse-transcriptase (RT) activity (Roche Colorimetric Reverse Transcriptase Assay, article 11468120910) which was put into relation to p24 content and international units based on the World Health Organization p24 reference standard (90/636). One IU/ml of this standards equals approximately 5.1 pg/ml of p24 (bioMérieux VIDAS HIV p24 II) and 0.0005 ng/ml of RT (Roche RT assay) (for more details see [4]).

### Sequence alignments

The Los Alamos National Laboratory database (http://www.hiv.lanl.gov/content/sequence/HIV/mainpage.html) was queried for all available HIV-1 p24 sequences (of any subtype). One sequence per patient (option in LANL database) was downloaded in aligned amino acid table format and the frequency analysis was conducted in Excel.

### Mutagenesis

Amino acid changes at position 16 and 170 in p24 were introduced by side-directed-mutagenesis of the VLP eukaryotic expression vectors, using the QuickChange II XL Site-Directed Mutagenesis Kit (200521, Agilent Technologies). Primers were as follows: pBV8-B-T16S 5’-GTACATCAGGCCCTATCACCTAGAACTTTAAATG-3’; pBV59-F2-T16S 5’-GTACATCAGCCTCTATCACCTAGAACTTTAAATG-3’ ; pBV11-B-S16T 5’-GTACATCAGGCCATAACACCTAGAACTTTAAATG-3’; pBV43-D-R170K 5’-GATTATGTAGATCGGTTCTATAAAACTCTAAGAGCCGAGC-3’; pBV60-D-R170K 5’-GATCGGTTCTATAAAACTCTAAGAGCC-3’; pBV42-12BF-N170K 5’-GACAGGTTCTTTAAGACCCTAAGAGCC-3’; pBV38-D-K170R 5’-GACTATGTAGATCGGTTCTATAGAACTCTAAGAGCCGAGC-3’. Mutagenesis was verified by sequencing (for sequencing primers see [4]).

### Diagnostic tests

VLPs were diluted to 10 IU/ml p24 antigen (or 0.005 ng/ml RT) in negative human plasma (Swiss Red Cross Blood Donation Centre Zurich) in a single batch per VLP and stored at −20 °C. Antigen reactivity of the (anti-HIV-negative) samples was analysed with the following CE-marked and FDA-approved diagnostic tests and platforms: Abbott Architect HIV Ag/Ab Combo; bioMérieux VIDAS HIV p24 II; Siemens Enzygnost Integral II; Siemens Enzygnost Integral 4.

### Western blot

VLPs in PBS were diluted in NuPage LDS sample buffer and reducing agent (NP0007 and NP0009, Life Technologies) to a final concentration of 10 ng/ml RT. Samples were heated for 10 min at 95 °C. SDS polyacrylamide gel electrophoresis was performed using 10 % gels in standard Tris/Glycine buffer, and proteins were blotted on nitrocellulose membrane by wet transfer over night. Primary mouse monoclonal antibodies used to detect HIV p24 were AG3.0 (4121, NIH AIDS Reagent Program) and YDHIV1gp24 (sc-73300, Santa Cruz). Polyclonal human anti-p24 plasma was obtained by pooling plasma from 10 HIV-positive individuals, inactivated with 0.5 % Triton X-100. Species-specific HRP-coupled secondary antibodies were obtained from KPL (goat-anti-mouse HRP 474–1806; goat-anti-human HRP 141–992). Super Signal West Pico (34077, Thermo Scientific) was used as chemiluminescent substrate and the reaction visualised in the ImageQuant LAS-4000 imager (GE Healthcare).

### VLP-ELISA

VLPs in PBS were lysed in 3x SNCR virus disruption buffer [5] and coated onto clear 384-well plates (Nunc MaxiSorb P6366, Sigma Aldrich) at a concentration of 5 ng/ml RT over night at 4 °C. After blocking with 2 % non-fat milk in PBS, primary and secondary antibodies (see Western Blot) were sequentially applied for 1 h with intermittent washes. TMB liquid substrate (T8665, Sigma Aldrich) was diluted at 1:2 in H_2_O and added for 15 min, followed by the addition of 1 M H_2_SO_4_ to stop the reaction. Optical density was measured at 450 nm on the Perkin Elmer EnVision microplate reader. Cut-off values were derived from wells mock-coated with the same reagent, but lacking antigen, and processed in the same way.

### Ethical approval

This study did not use any personal information, but only previously published viral sequences [4]. Therefore, in full accordance with Swiss legislation (Federal Act on Data Protection of 19 June 1992; see https://www.admin.ch/opc/en/classified-compilation/19920153/index.html) informed consent was not necessary.

## Results

### Amino acid variants

To identify amino acid variations which might be responsible for the failure to detect p24 in a diagnostic test, the p24 sequences of all 43 VLP panel members investigated in our previous study were aligned (sequences available at NCBI GenBank accession numbers KJ689249-KJ689290) [4]. This highlighted a single amino acid variation of Serine (S) to Threonine (T) at position 16 of pBV8-B and pBV59-F2; both VLPs had been the only ones poorly detected in two highly sensitive tests in our previous study (Siemens Enzygnost Integral 4 and Innogenetics Innotest HIV Antigen mAb). Furthermore, we identified a variation of Lysine (K) to Arginine (R) or Asparagine (N) at position 170, which might be accountable for the failure of the bioMérieux VIDAS DuoUltra and VIDAS HIV p24 II to detect VLPs pBV43-D, pBV60-D and pBV42-12BF. To ensure that amino acid variations in the Gag VLPs were not erroneously introduced during the Gag cloning procedure [4], the relevant part of the original viral RNA isolated from patient plasma was sequenced, and the amino acid variations at position 16 and 170 of the respective VLPs were confirmed at nucleotide level in the viral genome (data not shown).

In order to assess the frequency of the S16T and K170R/N variants in naturally occurring HIV-1 isolates, all available p24 sequences were downloaded from the Los Alamos National Laboratory database (http://www.hiv.lanl.gov/content/sequence/HIV/mainpage.html). Table 1 lists the total number of intact sequences at positions 16 and 170 and the frequency of amino acids at these positions grouped by common HIV-1 subtypes and CRFs. At position 16, 97.7 % (10’125) of all sequences had an S present. The second most common amino acid at this position was a T (174, 1.68 %), followed by A (62, 0.6 %). Interestingly, all of the 9 available HIV-1 group N sequences had a T at position 16. At position 170, 97.85 % (10’142) of all sequences had a K present. R accounted for the large majority of other amino acids at this position (216, 2.08), and an N was only found in three sequences (0.03 %).

**Table 1:** Frequency of amino acid variations for positions 16 and 170 of p24 of sequences deposited in the Los Alamos National Laboratory sequence database

Subtypes	Amino acids at position 16									Amino acids at position 170							
	S	T	A	H	L	M	Y	Q	Total	K	R	N	E	I	T	Y	Total
A (A1/A2)	330	6	2					1	339	334	3	1		1			339
	97.35 %	1.77 %	0.59 %					0.29 %		98.53 %	0.88 %	0.29 %		0.29 %			
B	4′387	78	45			1	1		4′512	4′412	112						4′525
	97.23 %	1.73 %	1.00 %			0.02 %	0.02 %			97.50 %	2.48 %				1		
C	2′369	50	11	2	1				2′433	2′357	68	1					2′426
	97.37 %	2.06 %	0.45 %	0.08 %	0.04 %					97.16 %	2.80 %	0.04 %					
D	123	5							128	126			1				127
	96.09 %	3.91 %								99.21 %			0.79 %				
F (F1/F2)	85	2							87	77	6	1					84
	97.70 %	2.30 %								91.67 %	7.14 %	1.19 %					
G	65	1							66	64							64
	98.48 %	1.52 %								100.00 %							
CRF01_AE	1′015	1	1						1′017	1′012	4						1′016
	99.80 %	0.10 %	0.10 %							99.61 %	0.39 %						
CRF02_AG	97				1				98	96	2						98
	98.98 %				1.02 %					97.96 %	2.04 %						
CRF07_BC	140								140	139							139
	100.00 %									100.00 %							
Other group M	1′456	20	3						1′479	1′457	20				1		1′478
	98.44 %	1.35 %	0.20 %							98.58 %	1.35 %				0.07 %		
Group O	20								9	20							20
	100.00 %									100.00 %							
Group N		9							20	8	1						9
		100.00 %								88.89 %	11.11 %						
Group P	2								2	2							2
	100.00 %									100.00 %							
Unclassified	36	2							38	38							38
	94.74 %	5.26 %								100.00 %							
Total	10′125	174	62	2	2	1	1	1	10′368	10′142	216	3	1	1	1	1	10′365
	97.66 %	1.68 %	0.60 %	0.02 %	0.02 %	0.01 %	0.01 %	0.01 %		97.85 %	2.08 %	0.03 %	0.01 %	0.01 %	0.01 %	0.01 %	

To investigate the impact of these amino acid variations on p24 detectability, we introduced a T16S change into pBV8-B and pBV59-F2, as well as an R170K change into pBV43-D and pBV60-D and an N170K change into pBV42-12BF. As control, we also introduced an S16T change into pBV11-B and K170R into pBV38-D, both VLPs having been detected well by all diagnostic tests previously investigated [4]. Table 2 summarises amino acids found in the “wildtype” VLPs as cloned from virus isolates, and changes introduced to investigate the impact of these amino acid variants on p24 detection, as described in the subsequent sections.

**Table 2:** Summary of VLPs investigated in this study

VLP	Accession number	AA position in p24 (Gag)^a^	AA in wt VLP	AA change introduced	HIV diagnostic test with low sensitivity for wt
pBV8-B	KJ689249	16 (148)	T	S	Siemens Enzygnost Integral II/4
pBV59-F2	KJ689286	16 (148)	T	S	Siemens Enzygnost Integral II/4
pBV11-B	KJ689251	16 (148)	S	T	N/A – control VLP
pBV43-D	KJ689277	170 (302)	R	K	bioMérieux VIDAS HIVp24 II
pBV60-D	KJ689287	170 (302)	R	K	bioMérieux VIDAS HIVp24 II
pBV42-12BF	KJ689276	170 (302)	N	K	bioMérieux VIDAS HIVp24 II
pBV38-D	KJ689274	170 (302)	K	R	N/A – control VLP

*AA* amino acid, *wt* wildtype, *N/A* not applicable as these VLPs were used as controls

^a^in HXB2 reference sequence; accession number in NCBI GenBank

### Diagnostic tests

**Fig. 1:**
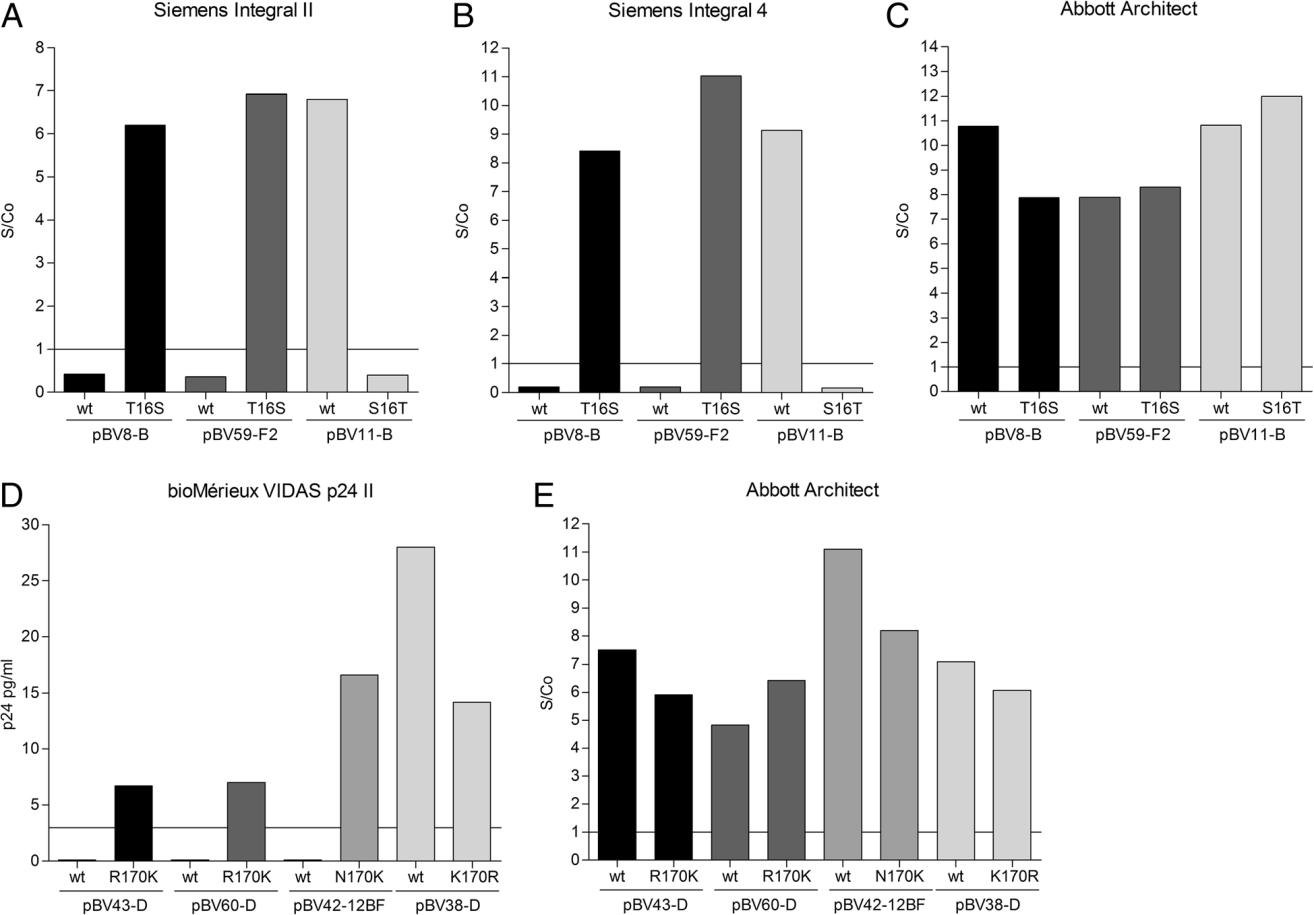
Detection of wildtype and mutated VLPs by diagnostic tests. VLPs were diluted to 10 IU/ml in negative human plasma and analysed with the indicated diagnostic tests. Panels **a**, **b** and **d** show antigen detection results of wildtype (wt) and mutated VLPs for those diagnostic tests which failed to detect wildtype VLPs harbouring the S16T (**a** and **b**) or K170R/N variant (**d**). Panels **c** and **e** show results of antigen detection by the Abbott Architect HIV Ag/Ab Combo to control for the presence of p24 antigen. Lines indicate S/Co = 1, which represents the lower limit of S/Co ratios for unequivocally positive samples according to the manufacturer’s instructions. In case of the bioMérieux VIDAS HIV p24 II the line indicates 3 pg/ml, denoting the lower limit of the quantifiable range for this test

All wildtype and mutant VLPs were diluted to 10 IU/ml p24 in HIV-negative human plasma and analysed using HIV diagnostic tests that had failed to detect the wildtype VLP variants (see Table 2). As control, these VLP preparations were also tested on the Abbott Architect HIV Ag/Ab Combo to confirm the presence of p24. As previously observed, the pBV8-B and pBV59-F2 wildtype VLPs were not detected by the Siemens Enzygnost Integral II and 4 at 10 IU/ml, whereas the positive control, wildtype pBV11-B, was detected well by both tests (Fig. 1a, b). In contrast, with the T16S mutation introduced, pBV8-B-T16S and pBV59-F2-T16S were both detected as well as pBV11-B, while the mutated control VLP pBV11-B-S16T was no longer detectable. The Abbott Architect yielded good S/Co ratios for all tested VLPs, confirming the presence of p24 in these samples (panel C). These results suggest a crucial role for the amino acid found at position 16 for the detection of p24 by the Siemens Enzygnost Integral II and 4.

Similarly, analysis of the position 170 wildtype and mutant VLPs on the bioMérieux VIDAS p24 II (Fig. 1d) showed a restored ability to detect the mutant pBV43-D-R170K, pBV60-D-R170K and pBV42-12BF-N170K. Introducing the amino acid variant (R) at position 170 into the wildtype pBV38-D, reduced, but not completely abolished, detection by the bioMérieux VIDAS HIV p24 II. The somewhat lower re-gained detectability of pBV43-D-R170K and pBV60-D-R170K and the only two-fold (rather than complete) loss off pBV38-D-K170R signal suggest that additional, unidentified epitope variations in these VLPs also play a role in detection by this test. Again, all VLPs were also tested on the Abbott Architect to control for the presence of viral antigen (panel E). These observations demonstrate that a single amino acid variation can account for the complete failure of p24 antigen detection by certain diagnostic HIV-1 tests.

### Western blot and ELISA

**Fig. 2:**
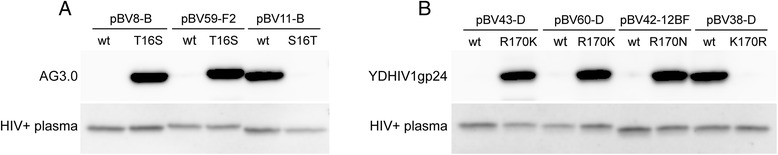
Detection of wildtype and mutated VLPs by western blot with epitope-specific antibodies. Position 16 and 170 wildtype and mutated VLPs were detected with the epitope-specific antibody AG3.0 (**a**) and YDHIV1gp24 (**b**), respectively. In parallel, the same sample preparations were loaded on a second gel and p24 was detected with pooled HIV+ plasma on the membranes to control for the presence antigen (**a** and **b** lower panels)

To further confirm the role of these single amino acid variations on antibody binding, the wildtype and mutant VLPs were analysed by Western Blot, using primary antibodies binding to the epitope-regions in question. The epitope of the antibody AG3.0 was mapped to the highly conserved motive SPRTLNA (position 16–22 in p24) [6] and antibody YDHIV1gp24 was largely mapped to amino acids 148–197 [7]. Figure 2 illustrates the inability of the respective antibodies to detect wildtype VLPs with the rare amino acids in the critical position and the restored detection upon mutation of these amino acids to the consensus sequences. Furthermore, introducing the amino acid variant into the consensus background abolished p24 detection of these VLPs (pBV11-B for AG3.0 and pBV38-D for YDHIV1gp24). On a parallel Western blot loaded with VLPs from the same sample preparation, p24 was detected using pooled plasma of HIV-1+ individuals, confirming the presence of p24 in all samples.

**Fig. 3:**
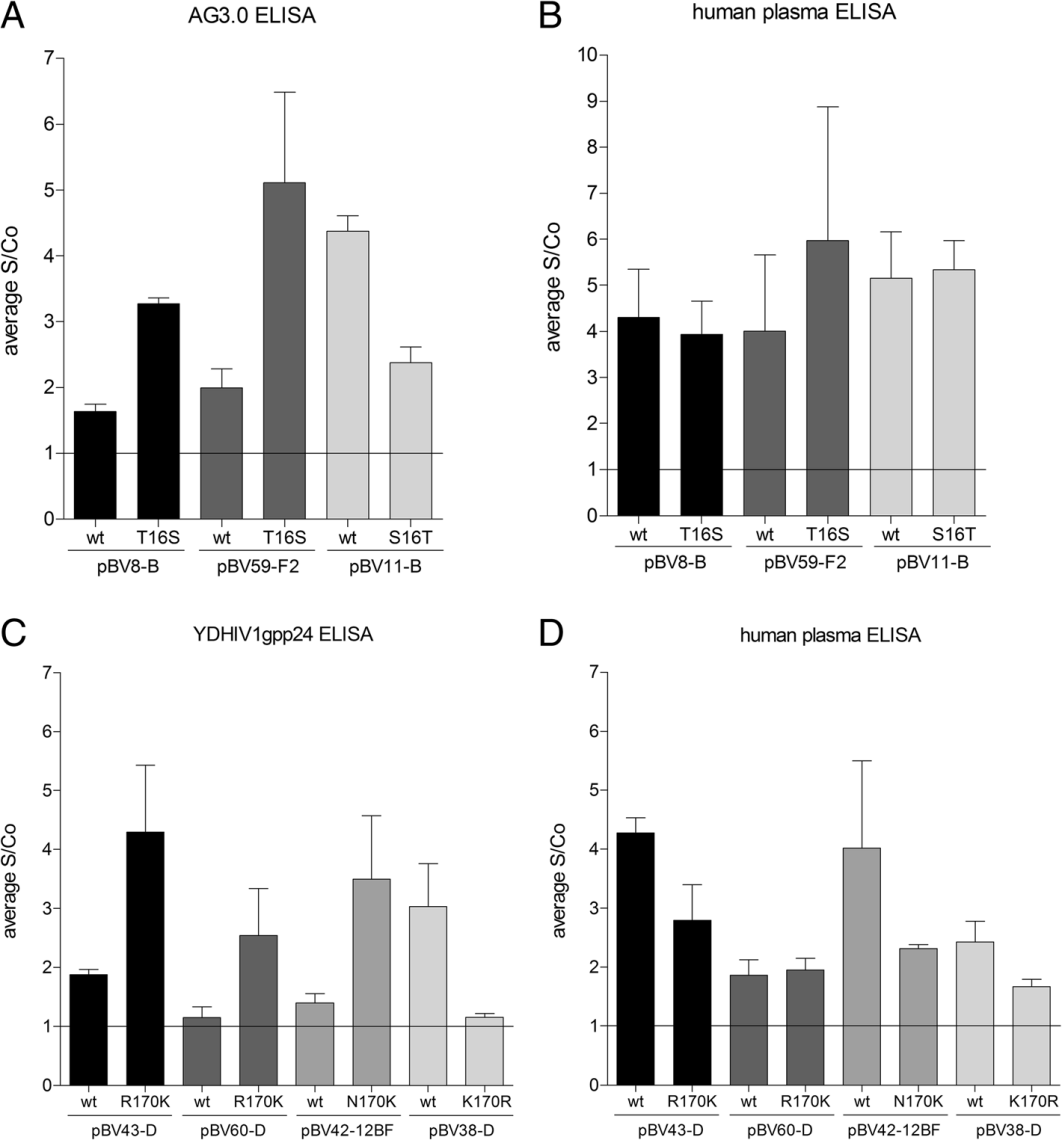
Detection of wildtype and mutated VLPs by ELISA with epitope-specific antibodies. Equal amounts of VLPs were lysed, directly coated on ELISA plates and detected either with epitope-specific antibodies (**a**: AG3.0 for position 16; **c**: YDHIV1pg24 for position 170) or pooled HIV+ plasma to control for the presence of antigen (**b** and **d**). S/Co ratios were calculated in relation to background signals of the respective antibodies on uncoated wells. Results are averages of three experiments. Lines indicate S/Co =1, above which samples were scored as detectable

In addition, we also tested the antibodies’ ability to detect p24 directly coated onto ELISA plates after VLP lysis. Figure 3 panels a and c show a reduced signal-to-cut-off (S/Co) ratio for the detection of the aberrant wildtype VLPs compared to VLPs mutated to the consensus sequence, and good detection of the control wildtype VLPs (pBV11-B and pBV38-D) compared to the matching mutated ones. All VLPs were also detected with HIV-1+ pooled plasma to control for the presence of p24 on the coated plates (panels B and C). Due to the high variability and background of this direct ELISA, results are not as clear-cut as for the Western blot, but they nevertheless support the observation that the investigated single amino acid variations can influence antibody binding to p24.

## Discussion

Modern HIV 4^th^ generation combination screening tests or antigen-only tests employed for HIV screening and diagnosis, are expected to detect all HIV-1 subtypes and circulating recombinant forms. Many of these tests currently on the market indeed have a fairly good sensitivity and subtype breadth [4, 8]. We were thus surprised that some otherwise highly sensitive tests failed to detect certain VLPs at a p24 concentration of 10 IU/ml, which roughly corresponds to 500’000 cp/ml RNA (for conversion see [4]). The analyses conducted in the present study suggest that this failure is not necessarily due to a set of subtype-specific amino acid sequence variations, but rather due to single amino acid variations. The amino acids at position 16 and 170 identified here as being responsible for detection failure, are both located in highly conserved regions of p24 and deviations from the consensus sequence are of conservative nature. Although position 16 is part of the well-studied HLA-B*57-restricted CTL epitope ISW9 [9] and hence target of selective pressure, escape variants have only been described for positions 14 and 15 [10, 11]. It’s role might be rather of structural nature, as position 16 is part of an N-terminal three amino acid linker-region (14–16) in p24 [12], which plays a role in protein re-folding upon proteolytic processing of Gag [13]. Position 170 is part of the major homology region (MHR) of p24 (153–172), the most conserved part of the viral capsid found in many retroviruses, with an essential role in Gag assembly [14, 15] and viral infectivity [16]. The rather conservative nature of the K to R variant and the relative low frequency of less conservative substitutions at position 170 are in line with the importance of conserving the MHR function. An Alanine exchange at K170 was found to maintain assembly function but not infectivity of viral particles [16, 17]. Notably, a certain degree of flexibility seems to exist, given the K to N change (relatively large positive to smaller uncharged properties) observed in pBV42-12BF. It is unknown if this requires compensatory second-site changes in the protein.

The frequency of S16T and K170R variations found in the LANL deposited sequences, suggests that as many as 1:50–1:60 patients carry these variations. While it is not known how frequent they are in primary infection, their importance in the use of immunoassays should be kept in mind when selecting epitope regions for generation of antibodies for diagnostic tests. Our previous study suggests that aside from the Siemens Enzygnost Integral II and 4, the Innotest HIV Antigen mAb from Innogenetics also targets position 16. Here too, pBV8-B and pBV59-F2 were the only VLPs of the whole panel to be poorly detected [4]. Furthermore, in addition to the bioMérieux 4^th^ generation combo test (DuoULTRA) and antigen-only test (HIV p24 II), the Siemens ADVIA CHIV also had poor sensitivity for pBV43-D, pBV60-D and pBV42-12BF, indicating binding of antigen around position 170 in this test. Notably, the bioMérieux tests detected several VLPs with severely lower sensitivity [4], but obvious amino acid variations could only be identified for the three VLPs carrying the position 170 amino acid variant. This suggests that other epitopes targeted by this test are also sensitive to sequence variation.

## Conclusion

Although both amino acid variations described in this study are of rather conservative nature, their impact on antibody binding and detection of p24 by diagnostic HIV tests is striking. It demonstrates that test sensitivity and breadth of detection are not only defined by subtypes and their associated overall sequence variations, but also by single amino acid polymorphisms. Future antigen test development should take these findings into account to cover the broadest spectrum of circulating isolates possible.
